# Extending the theoretical framework for curriculum integration in pre-clinical medical education

**DOI:** 10.1007/s40037-017-0348-y

**Published:** 2017-04-07

**Authors:** John Vergel, Diana Stentoft, Juny Montoya

**Affiliations:** 1 Centro de Investigación y Formación en Educación (CIFE), Universidad de los Andes, Bogotá, Colombia; 2 Centre for Health Science Education and Problem Based Learning, Department of Health Science and Technology, Aalborg University, Aalborg, Denmark

**Keywords:** Curriculum design, Curriculum integration, Problem-based learning, Grounded theory, Medical education

## Abstract

**Introduction:**

Curriculum integration is widely discussed in medical education but remains ill defined. Although there is plenty of information on logistical aspects of curriculum integration, little attention has been paid to the contextual issues that emerge from its practice and may complicate students’ knowledge integration. Therefore, we aimed to uncover how curriculum integration is manifested through context.

**Methods:**

We collected data from the official curriculum and interviewed ten participants (including curriculum designers, facilitators, and students) in the bachelor’s medical program at Aalborg University. We observed various learning activities focused on pre-clinical education. Inspired by grounded theory, we analyzed the information we gathered.

**Results:**

The following theoretical constructs emerged after the inductive analysis: 1) curriculum integration complexity is embedded in the institutional learning perspectives; 2) curriculum integration is used to harmonize conflicting learning perspectives in curriculum practice; 3) curriculum integration creates tensions that self-organize its structure; and 4) curriculum integration becomes visible in collaborative learning spaces.

**Discussion:**

These constructs provide a framework for analyzing curriculum integration in the context in which it is meant to appear, which may assist educationalists to gain a more specific understanding of the term. This may enable effective curriculum integration since contextual issues are addressed in addition to the goals specified in the official curriculum.

## What this paper adds

Curriculum integration is considered of key importance for reforming medical programs across the world, yet many medical schools struggle with integrating their curricula. This is possibly a consequence of the confusion derived from diverse definitions of curriculum integration anchored in multiple learning theories. Moreover, the existing definitions pay little attention to the contextual issues of medical schools. Approaching curriculum integration through an extended theory that takes into consideration contextual issues may provide medical educators insights into contextually determined conflicts, tensions and learning perspectives influencing the curriculum practice.

## Background

Curriculum integration has been widely recommended in the field of medical education, although the concept remains ambiguous [1–9]. Curriculum integration facilitates learning by helping students to see the complete curricular picture, as well as its underlying connections; thus, students do not merely memorize separate topics, but understand information in a comprehensive manner [10]. Nevertheless, the meaning of curriculum integration is unclear, possibly owing to the multiple variations of it that include the following:

- Integrating basic and clinical sciences [11];
- Integrating basic, clinical, and social sciences [12];
- Integration based on delivering information [8];
- Integration based on applying prior knowledge and experience [13];
- Deliberately unifying separate areas of knowledge in the curriculum [5];
- Interacting knowledge derived from multiple sources to foster understanding and performance of medical activities [14];
- The ‘dynamic interconnectedness that emerges from recursive interactions at multiple levels’ [15];
- Designing modules that have ‘an overall theme which governs the horizontal integration of all relevant disciplines’ [16]; and
- An ‘iterative revisiting of topics, subjects or themes throughout the course’ [17].

These multiple approaches for integrating curriculum are based on different learning theories that provide underlying organizing principles such as discipline-based learning, cognitivism, behaviourism, constructivism, complexity theory, and spiral learning [18–20]. The lack of consensus on the understanding of curriculum integration is confusing for those who deal with its practice. We speculated that this theoretical ambiguity of curriculum integration stimulates the considerable attention given to the curriculum structure, instead of a focus on how and why learning theories facilitate the curriculum connections [21–23]. This instrumental approach further muddles the understanding of curriculum integration practices as it pays limited consideration to contextual issues, such as having different learning perspectives or a hidden curriculum. In establishing our earlier definition of curriculum integration prior to this study and in the light of the meanings described above, we missed an extension of the curriculum integration theory that also considers the issues of its actual practice. Consequently, we sought ways to include these issues in our theoretical conception of curriculum integration by examining it contextually, since the concepts of integration and curriculum are embedded into the specific educational context. Therefore, we investigated how students, facilitators, and curriculum designers understand and operationalize curriculum integration in a particular setting [24].

We conducted a case study in the medical program at the School of Medicine and Health at Aalborg University in Denmark. The following research questions guided our study: 1) What is the program’s official perspective on curriculum integration? 2) What do curriculum designers, facilitators, and students understand by ‘curriculum integration’? 3) How is curriculum integration manifested in everyday teaching practice and learning activities?

## Methodology

### Research paradigm

This paper reports on qualitative research, specifically a case study, in which we applied some of the inductive analytic techniques that are commonly used in grounded theory to interpret data. We thought about constructing a theoretical framework of curriculum integration grounded in the idea that research participants define social actions, such as the curriculum practice [25]. From this, we can develop a broader theory that includes contextual issues on the participants’ definitions of their curriculum practice.

### Setting

According to the official curriculum, Aalborg University’s medical education program offers a compounded, three-year, pre-clinical bachelor’s degree and a three-year clinical master’s degree. The learning activities are grounded in the problem-based learning (PBL) ‘seven jump’ model [26], and learning goals (as set forth in the curriculum) are organized around a specific set of knowledge, skills, and competences. Moreover, the institutional learning perspectives are anchored in PBL. The PBL curriculum is implemented through various activities such as case-oriented PBL, project-oriented PBL (developing skills in collaborative work and project management), supporting lectures, resource sessions, clinical exercises, and clinical practice.

As follows, we describe in more detail the program theory that underlies this curriculum planning and the expected processes, outcomes, and goals relating to integration. Integration is expected to occur at different curriculum levels. At the macro levels of curriculum integration, students are expected to develop knowledge of the human organ systems (such as the musculoskeletal, nervous, and gastrointestinal systems); this is embedded in the integration of knowledge, skills, and attitudes from the angle of basic/biomedical, clinical, and social sciences (such as person-centred care, life sciences, managing resources, immunology, population health, and evidence-based medicine). At the same levels, it is also expected that certain topics (i. e. adrenergic receptors) will be addressed at least twice as the program progresses from dealing with the body’s normal state toward the disease state. With this curriculum design, integration aims for students to achieve a deeper and more complex understanding each time they revisit the organ systems.

**Fig. 1:**
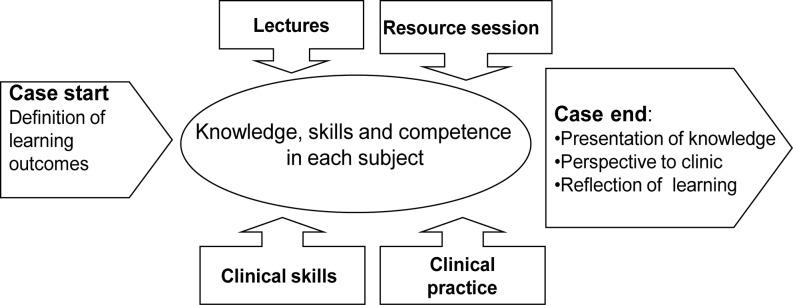
Official curriculum integration. Figure kindly provided by Dr. Trine Fink, associate professor, Department of Health Science and Technology, Aalborg University, and Dr. Jeppe Emmersen, associate professor, Head of School of Medicine and Health, Aalborg University

In addition, as shown in Fig. 1, the micro levels of curriculum integration are expected to connect various learning activities that are intended to link students’ prior knowledge with the new information addressed via an inquiry process. Cases constitute the major learning activities, whereas resource sessions, lectures etc. are intended to support students’ case work. In the case activities, 10 to 14 students discuss a patient’s clinical information guided by a facilitator. The patient’s history was unstructured, incomplete, and included social issues. The students are expected to go through seven phases in accordance with the seven-jump model: five during case opening, step six is their learning in between the opening and closing of the case, and step seven is the case close sessions.

### Design

In the study we adopted a multi-method approach to obtain a complete description of curriculum integration at the pre-clinical bachelor program [27]. At the design stage of the study, we were concerned about how we could avoid arbitrarily imposing our subjectivity on our interpretations of the data; we wanted to minimize our biases in recruiting participants and interpreting their subjective experiences. That is why we decided that Researcher 1, who did not work or study at Aalborg University, should collect the qualitative information and recruit participants. The steps described in the Methods section were conducted over a six-month period. We chose Aalborg University because it implements a specific curriculum integration approach anchored in PBL, which intrigued us due to its role in fostering integrated knowledge. Moreover, we decided to analyze the official curriculum, which was delivered in English, before Researcher 1 observed the learning activities to understand the actual operationalization of curriculum integration in practice. Researcher 1 collected the data. Researcher 2 is a senior researcher in PBL at Aalborg University, and Researcher 3, who is a senior investigator at Universidad de los Andes, Colombia, where follow-up studies were planned, contributed to the subsequent data analysis.

**Fig. 2:**
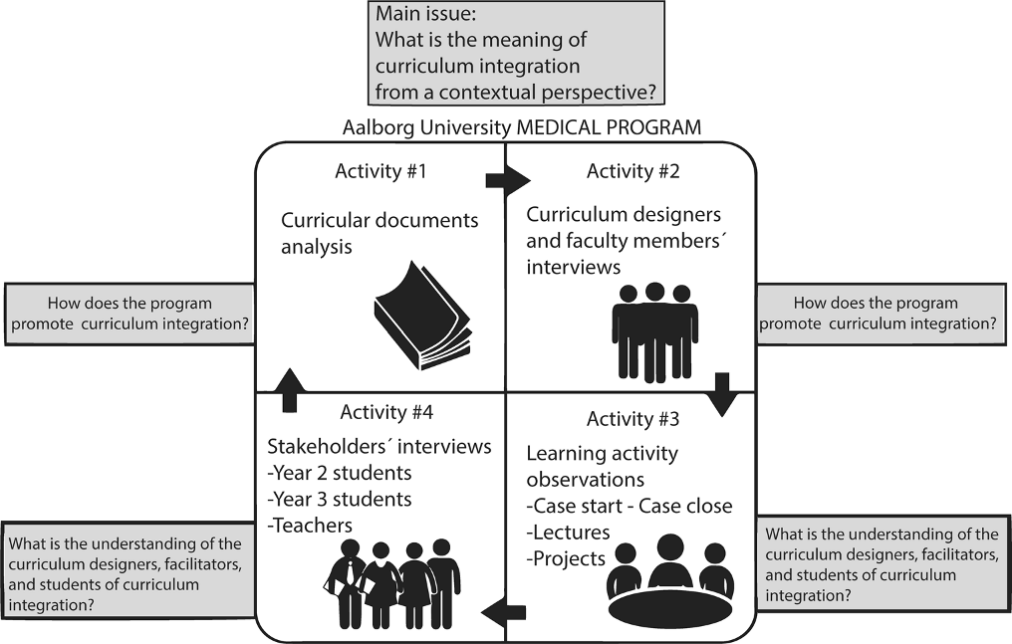
Research design. *CI* curriculum integration, *AAU* Aalborg University

As shown in Fig. 2, the design included four stages. First, for document analysis, we studied the official curriculum to identify the structure of curriculum integration, the type of curriculum, and the connections among its elements. Second, Researcher 1 interviewed three curriculum designers (in English) to understand the rationale behind curriculum integration. These interviews were semi-structured and had open-ended questions (Table 1). Third, Researcher 1 observed nine learning activities in the musculoskeletal and gastrointestinal modules, in the 2nd and 3rd year. In the observations, Researcher 1 aimed to determine how the curriculum was implemented. The non-participatory and non-blinded observations were primarily focused on PBL cases, and Researcher 1 strategically selected the observational units before conducting observations by asking the following questions:

1. What do students learn?
2. How are students learning it?
3. What knowledge is integrated and how are students integrating it?

**Table 1:** The prompting questions for the semi-structured interviews for each group

*I*	*Curriculum designers*	
	A	What is the meaning of ‘competences’ in the curriculum?
	B	What is the meaning of ‘research-based program’?
	C	What is the difference between competences and skills, and between clinical skills practice and actual clinical teaching?
	D	Why does the program implement PBL with cases and projects? What is a case and what is a project?
	E	Why do the students learn about all organs at least twice (cyclic learning)?
	F	What do you expect of projects in conjunction with international partners?
	G	What is a portfolio?
	H	How do students implement new technology in the treatment of medical diseases? Where this does occur?
	I	How are the basic clinical sciences integrated in the modules?
	J	How are the normal-abnormal human body functions integrated in the modules?
	K	Why do medical students need to learn basic sciences?
*II*	*Facilitators*	
	L	What is your role as a case facilitator?
	M	What is the purpose of identifying keywords and connecting them using arrows?
	N	How do you know that your students are really learning?
	O	If medical books have a lot of information that students need to read and study, how do you manage that in one week?
	P	Why do some students participate little in the discussions?
	Q	What is the difference between the start and close of a case?
	R	How do you know that your students are integrating knowledge?
*III*	*Students*	
	S	What and how do you do in cases?
	T	What is a keyword?
	U	What is the purpose of identifying keywords and connecting them using arrows?
	V	How do you connect the separated information in the fields of anatomy, pathology, and histology?
	W	How do you know what you have to learn?
	X	What is a lecture about? What does the professor do?
	Y	What do you do when a case is closed?
	Z	How do you know that you are learning?

Researcher 1 took field notes, but the main challenge was the language. Although the students spoke English fluently, they preferred conducting discussions in Danish, whereas the facilitators guided them in English. As Researcher 1 did not speak Danish, he performed *post hoc* interviews with two students in English to fully comprehend the discussions during the case session. Finally, Researcher 1 interviewed two facilitators and five students in regards to their interpretations of the learning activities. These interviews were also in English, semi-structured, and with open questions, as shown in Table 1. The participants signed an informed consent form before data collection began. All interviews were audio recorded and transcribed for further analysis. Importantly, interviews and observations were made anonymous. Given the nature of the study, approval from an ethical committee was not necessary as per the regulations of the institution.

### Participants

The ten participants consisted of three curriculum designers, two case facilitators, and five students from two separate study groups in their 2nd and 3rd year of the pre-clinical bachelor program. We selected participants as a ‘convenience sample’ using the technique of ‘snowball sampling’ [25]. Since we aimed to construct a theory transferable to other settings, we gathered data until reaching saturation, described below as an inductive analysis, then developed the theory based on the repeating information provided by the ten participants [28].

### Data analysis

We performed an inductive analysis [29]. We started transferring the text to the software program ATLAS.ti (Version 6.2). Then, we repeatedly read the raw information and selected segments relevant to our research questions. In the follow-up phases, we interpreted these segments into themes. Next, we arranged the themes into groups (based on their logical connections with the reviewed literature [Table 2]) and interpreted the groups by categorizing them into theoretical constructs.

**Table 2:** Theoretical constructs, themes^a^, and sources of data

*I*	*CI complexity is embedded in institutional learning perspectives*		
	A	There are many types of organizational elements in the curriculum that are connected in varied, complex ways	
		1	Curriculum designer
	B	Organ systems integration	
		1	Official curriculum
		2	Observations
	C	Humanities-clinical sciences integration	
		1	Official curriculum
	D	Spiral integration	
		1	Official curriculum
		2	Observations
	E	Cases are successfully used when students read them, brainstorm, derive knowledge, and report on each case in a one week cycle	
		1	Curriculum designer
		2	Observations
*II*	*CI is used to harmonize conflicting learning perspectives in curriculum practice*		
	F	Experience-topics integration in cases	
		1	Curriculum designer
		2	Facilitator
	G	Disciplinary knowledge has an important influence in the learning outcomes of the official curriculum	
		1	Official curriculum
		2	Curriculum designer
	H	There are some tensions in the curriculum related to the university’s learning perspectives and the broad topics in medicine	
		1	Observation
		2	Student
*III*	*CI creates tensions that self-organize its structure*		
	I	Tension between students’ expectations and the official documents of the study program	
		1	Facilitator
		2	Observation field notes
	J	Students adjust their learning activities when they struggle with the sessions	
		1	Student
*IV*	*CI becomes visible in collaborative learning spaces*		
	K	Case close sessions enable students to discuss and explain their learning outcomes	
		1	Facilitator
		2	Student
	L	Drawing on the blackboard is an activity that supports learning	
		1	Observation field notes
		2	Student

^a^The table shows some of the themes from which constructs emerged, and these themes were chosen arbitrarily from the theme lists to better represent the inductive analysis

We organized the analysis as a cyclical interpretation of the data, meaning that the theoretical constructs were continuously re-elaborated after multiple sessions of discussion and reflection among Researchers 1, 2, and 3, and between the researchers and participants, such as the curriculum designers. In addition, to clearly illustrate the process of analysis, in Table 2 we arbitrarily depict the themes that provided the most relevant information toward creating the theoretical constructs. Each theoretical construct had between 6 and 24 themes and 33 to 84 quotes.

### Credibility of the interpretations

We used proven strategies to explain how our constructs emerged from the data instead of our biases [30]. Rubin and Rubin [30] provide criteria for using subjectivity in research analysis in a justifiable manner. We applied such criteria, including 1) *transparency* (we showed how we interpreted the data to other groups of researchers in the field of education); 2) *communicability* (we discussed if our constructs made sense with other researchers), and 3) *coherence* (we checked whether our constructs told the reader a coherent story).

Furthermore, while developing the theoretical constructs, we used triangulation to examine if our interpretations were found in multiple sources [31]. For example, as shown in Table 2, we discovered data in the observations, as well as in the interviews with students and facilitators, which led us to infer that integration of knowledge was visible in collaborative learning activities (the fourth construct). The data included: 1) Descriptions of how a facilitator identified with learning assessments when students integrated knowledge; 2) How a student acknowledged that their classmates were making connections between learning outcomes; and 3) Field notes on students’ discussions about the conceptual maps they drew on the blackboard in PBL sessions. These pieces of evidence indicated to us that the interpretations were justifiable.

## Results

We identified four theoretical constructs. The following relates the participants’ curriculum integration experiences when seen in light of the four theoretical constructs.

### Curriculum integration complexity is embedded in the institutional learning perspectives

The curriculum designers perceived curriculum integration as a complex structure; that is, multiple curriculum elements are integrated at both the macro and micro levels of the curriculum structure. In curriculum design, many learning activities (the micro level) must interact for students to attain varied learning outcomes from diverse disciplines (the macro level). For example, lectures and theoretical exercises are linked to PBL cases to support students’ learning of biomedical and clinical knowledge, social issues (the ‘soft learning’ outcomes, such as the influence of loneliness on healing), and technology-related topics (such as the impact of technology on health systems).

However, in this case, the challenge is how the curriculum elements need to be integrated based on certain principles in order to successfully handle such a complex curriculum integration approach. As such, by using the collected data, we deduced that the institutional learning perspectives guide the integration. For instance, institutional learning perspectives are understood as PBL grounded in constructivist thinking and experiential learning. Hence, curriculum integration is organized to support specific ways for students to establish their knowledge, as described in the following excerpt:

(…) this week there are a number of activities that they [students] could engage [in], so we have some lectures. Lectures do not give them the answer to the learning objectives (…) the lectures [give] an overview of a broad topic (…) [to] give the students ideas about where they [could] go and seek out more resources.

(Curriculum designer S)

Consequently, the institutional learning perspectives (that is to say, the way that students are intended to construct knowledge) orient the way that learning activities are integrated in curriculum design, including how activities are supposed to achieve learning outcomes in an integrated manner.

### Curriculum integration is used as a tool to harmonize conflicting learning perspectives in curriculum practice

The curriculum designers also mentioned that they had experienced curriculum integration where conflicts occurred. Although a curriculum designer noted that ‘(…) the curriculum is not defined in a traditional way where the curriculum [focuses on] books and [the] pages of books’, traditionally, medical education has been influenced by learning disciplinary topics (i. e., through lectures or individual readings of textbooks). Thus, curriculum designers not only needed to integrate activities framed on constructivism – otherwise known as experience-based integration [31] – but also on disciplinary topics, or subject-based integration [31].

To mitigate the negative effects of this tension, which was an issue in this case, curriculum designers established learning activities for each integration approach, such as students’ collaborative discussion of clinical cases for experience-based integration and lectures for subject-based integration. Designers then connected these learning activities to both experiences and subject-based integration approaches – using curriculum integration as a strategy to support students’ learning. The curriculum facilitated this high-level integration in the case-oriented PBL activities. The following quote captures this intention:

(…) In cases [in general], students define what they need to gain knowledge, and they can use the lectures as [input for] the case, and they can also use their experience in clinics, and they can use their books, or they can use their experiences in theoretical exercises. So, they can discuss [all this knowledge] in cases [in general] (…)

(Curriculum designer Z)

### Curriculum integration creates tensions that self-organize its structure

The students in our study mentioned that several tensions emerged from the curriculum integration practice. They believed the tensions arose when the institutional learning perspectives framing curriculum integration did not correspond to their learning expectations. One student provided an example:

(…) Sometimes, I choose not to go to the lectures because they only last 45 min, so they have to focus on some things and not talk about other things. Thus, I do not get what I need in the lectures every time.

A number of students saw lectures as unnecessary, or had different expectations of how these learning activities should be organized:

(…) I think it is really nice to have just one lecture (…) before we have the case start. Thus, we have a bit more knowledge, and the brainstorm[ing] is better because when we look at a case, we see [it in the way that] the (teacher) [says] something about osteoporosis or something.

(Student Y)

Due to these tensions, the participating students explored alternative learning activities integrated into the major curricular components. They changed the structure of curriculum integration during the curriculum practice due to the tensions they perceived in their learning. Therefore, the tensions self-organized curriculum integration by including new curriculum elements, as shown by the following excerpt:

I’m very visual and I have to see stuff in drawings. [For example] If you would like to explain how a bone looks [then they need to] have a Power Point presentation. Which is really good. But sometimes, they don’t explain things in a simple way … they say a lot of unnecessary stuff [such as] history. (…) Instead, I like to watch … videos on the Internet, and read my books, and then I write my stuff down in my notebook. When I’m done, and when I have written down what I learned, I go to the lecture, and I can see [what the teacher is talking about]. [I tell myself] Okay, I have to learn this. Okay, I got it.

(Student W)

### Curriculum integration becomes visible in collaborative learning spaces

The facilitators noted that the students were integrating knowledge when jointly discussing the cases. When Researcher 1 asked a facilitator how he realized that the students were learning, the facilitator responded:

(…) well, it is not easy to see the students’ brains, but somehow I realize when they ‘make a click’ [students give a knowing look] at some point during the brainstorming session. When they start discussing objectives, (…) they can realize the connection between different things and say ‘Okay, that makes sense.’ At that point, I can see not that they are learning, but that they are making sense [of the material] and connecting different things.

(Teacher P)

In his observations, Researcher 1 also found that collaborative learning activities were suitable venues for identifying the effectiveness of curriculum integration in promoting knowledge integration. In these activities, students could explain their understanding of the topics by themselves and to one another. For instance, students discussed issues using information from multiple disciplines, and explained what they learned based on the issues.

## Discussion

As educationalists, we face challenges such as helping students to connect diverse elements of disciplinary knowledge more easily than in traditional education, where knowledge integration was implicitly a task for learners. For example, the curriculum explicitly includes an examination of how integrating information from basic and clinical sciences might boost students’ healthcare practice, as patients’ clinical problems may be better understood using a biomedical basis [32, 33]. However, curriculum integration theories play little attention to contextual practice issues that may complicate students’ knowledge integration. Thus, we aimed to use our case study to construct an analytical framework that considers such issues. The findings revealed four theoretical constructs that frame curriculum integration as a dynamic, complex process highly dependent on context.

First, the construct *Curriculum integration complexity is embedded in institutional learning perspectives *implies that integration is a complex process that will be challenging for students to understand if a large number of topics from multiple disciplines (at different curriculum levels) are connected. This issue could be addressed by using the institutional learning perspectives to explain to students how they can visualize these connections. Since curriculum integration is embedded in learning perspectives, students might more easily understand why some (or which) learning activities, topics, and disciplines (i. e., curriculum components) are integrated with learning perspectives. For instance, resource sessions occurred in our study after the students had begun to investigate cases, not before, because Aalborg University views learning as an inquiry process. Students identify the learning outcomes they need to investigate before engaging in subsequent connected yet activities, including resource sessions. In this way, knowledge integration from the time a case is first examined, as well as during resource sessions, may be enhanced as students can visualize the curriculum structure and understand the reasons behind connections.

Muller et al. [34] suggested that curriculum integration should be understood as a complex process with multiple connections. Nevertheless, many medical education researchers have reduced curriculum integration to a connection between basic and clinical sciences, leaving aside many other relevant curricular interrelationships [34–40]. This reductionist approach may negatively affect how integrated curricula are understood. Since such an approach emphasizes biomedical-clinical dualism, it is not possible to consider curriculum complexity in the light of existing different connections.

The second and third constructs (*Curriculum integration is used to harmonize conflicting learning perspectives in curriculum practice *and* Curriculum integration creates tensions that self-organize its structure*) represent the dynamic nature of curriculum integration in practice. By ‘dynamic’ we mean curriculum integration mitigates tensions that may emerge from the interaction of diverse institutional learning perspectives but, simultaneously, produces tensions between the learning perspectives and the stakeholders’ learning expectations. In our case, the second construct refers to the tensions that emerged from the experience- and subject-based integration approaches. This issue is framed by the belief that studying medicine implies constructing knowledge from authentic learning experiences while also focusing on medical subjects, which entail different educational goals. This construct implies that curriculum integration may harmonize conflicts arising from the interaction of diverse learning perspectives. For example, case activities (the logistical basis of curriculum integration) combined subject- and experience-based integration by using collaborative discussions (experiences) about clinical issues to explore broad topics (subjects). Therefore, the interaction of two different educational goals with different pedagogical approaches (which may cause tensions in practice) can be harmonized by integrating them into the curriculum via a learning activity that is grounded in institutional learning perspectives (inquiry processes and PBL).

The third construct shows that curriculum integration does not remain static; instead, its structure changes during practice. In our case, curriculum integration practice led to disorganization in the curriculum owing to students’ specific learning expectations. When students approached institutional learning perspectives with their expectations, the pre-established curriculum arrangement was challenged and distorted, creating new and emerging patterns of connections, such as watching videos instead of attending a lecture. We interpreted this phenomenon as self-organizing integration, a characteristic of curriculum integration in which new patterns are created as a result of feedback produced during curriculum implementation [41]. We posit that self-organization as a characteristic of curriculum integration in the design, implementation, and evaluation of integrated curricula might contribute to improving knowledge integration. Since curriculum evaluation would bring to light the tensions arising from integration, it might be possible to undertake specific efforts to resolve such issues.

The fourth construct *Curriculum integration becomes visible in collaborative learning spaces* denotes the link between curriculum integration and students’ knowledge integration. Knowledge integration was explicitly demonstrated in students’ informed explanations of topics during learning activities. This outcome could be interpreted as the result of learning through curriculum integration, especially in social learning sessions. Therefore, this construct contains clues about how to evaluate the effectiveness of curriculum integration in promoting knowledge integration. Understanding curriculum integration beyond its logistical function is to characterize it as an educational aim [14, 42], which entails achieving ‘a conceptual, cognitive connection between different types of knowledge’ [43]. Knowledge integration was displayed in the collaborative learning sessions that our research subjects participated in; therefore, evaluation could usefully be focused on these sessions to provide feedback on how students integrate knowledge.

This study has some limitations that must be considered when estimating the transferability of our theoretical constructs about curriculum integration to other medical education contexts. Although Auerbach and Silverstein [29] claim that theoretical constructs could be extended beyond a particular setting, we acknowledge that further research is needed to explore how our constructs could apply to different medical education cultures or types of integrated curricula. Moreover, the constructs need to be refined by new challenges; for example, using the constructs as categories to understand participants’ descriptions of their curriculum integration experiences or elaborating them based on new contextual issues identified through analysis. The applicability of our constructs should be evaluated in various medical schools and in the clinical part of medical education, where the integration of knowledge takes a much more dynamic and less predictable form.

## Conclusions

The theoretical constructs presented in this study provide a broader understanding of curriculum integration because they take into account the challenges that medical schools may face when integrating their curricula. As challenges emerge from contextual issues, the constructs may be a framework for analyzing the practice of curriculum integration more closely and accurately. We believe, based on this framework, that addressing curriculum integration issues requires a profound shift away from an instrumental, static, reductionist perspective towards thinking of curriculum integration as a dynamic, complex process that may be influenced by all stakeholders involved.
